# DEMYSTIFYING IMPACTS OF NEIGHBORHOOD ENVIRONMENT ON HEALTH OF OLDER CHINESE AMERICANS

**DOI:** 10.1093/geroni/igad104.1407

**Published:** 2023-12-21

**Authors:** Yanping Jiang, Fengyan Tang

## Abstract

Residential segregation is a key social determinant of health that disproportionately affects racial/ethnic minority and immigrant populations. The health impact of residential segregation and subjective neighborhood environment, however, is poorly understood among older Asian Americans. Findings from the limited existing studies on this topic exclusively relied on cross-sectional data and/or masked distinctive differences across Asian subgroups due to the usage of aggregated data. The purpose of this symposium is to provide a better understanding of health impacts of residential segregation and subjective neighborhood environment among older Chinese Americans, the largest Asian subgroup in the U.S. Findings presented in this symposium were from the Population Study of Chinese Elderly in Chicago (PINE) study, a longitudinal study of community-dwelling older Chinese immigrants living in greater Chicago. Session 1 describes key characteristics of neighborhood, sociodemographic, and health behaviors of older Chinese immigrants living in segregated neighborhoods. Session 2 moves beyond to determine unique and joint effects of residential segregation and neighborhood socioeconomic status on 8-year change in cognitive functioning. Session 3 focuses on mental health by investigating main and indirect effects of residential segregation on depression via social processes. Session 4 tests the interactive effects of social support with residential segregation on cognitive functioning and depression. Lastly, Session 5 demonstrates how subjective neighborhood environment, directly and indirectly, affects depression via coping. In sum, findings provide innovative and important evidence to facilitate a better understanding of health implications of living in segregated neighborhoods among older Chinese Americans.

